# A Commentary on: “A New Removable Uterine Compression by a Brace Suture in the Management of Severe Postpartum Hemorrhage”

**DOI:** 10.3389/fsurg.2015.00017

**Published:** 2015-05-13

**Authors:** Shigeki Matsubara, Hironori Takahashi, Alan K. Lefor

**Affiliations:** ^1^Department of Obstetrics and Gynecology, Jichi Medical University, Tochigi, Japan; ^2^Department of Surgery, Jichi Medical University, Tochigi, Japan

**Keywords:** B-Lynch, postpartum hemorrhage, removable suture, uterine atony, uterine compression suture

Aboulfalah et al. (1) introduced a unique technique for a uterine compression suture (UCS), which included suture removal within 48 h after delivery. This technique is epoch-making.

To obstetricians, the year 1997 is memorable regarding the treatment of postpartum hemorrhage (PPH), when B-Lynch et al. introduced the UCS (2), which dramatically changed the treatment of PPH from hysterectomy to uterus-conserving UCS. During the last two decades, up to 15 modified UCS techniques have been published; we introduced the Matsubara–Yano (MY) UCS (2). The hemostatic effectiveness of the UCS is well established (2).

Uterine compression suture is not without side effects, including uterine necrosis, synechia, and infection (2). The UCS, by apposing the anterior and posterior uterine walls with a tied suture, necessarily limits blood flow to the uterus, which may cause uterine ischemia. The UCS remains in the uterine cavity until it is absorbed, which may lead to uterine infection. In either scenario, the suture is the culprit. Since the incidence rate of these adverse events is considered low, leaving the suture in place is considered a “necessary evil” for this life-saving procedure (2). The uterus usually contracts within a short period of time after delivery, resulting in hemostasis, and, thus, the “critical period” requiring the hemostatic effect of the UCS may be only 24–48 h after delivery. After this period, uterine compression may no longer be needed. Since the suture is responsible for adverse events and since uterine compression may be no longer needed after 48 h, removing the suture after 48 h may reduce the incidence of complications. Aboulfalah et al. (1) reported this approach, and that is why we consider their technique as epoch-making. Here, we offer clarification and concern.

Clarification is needed regarding the technical procedure. The explanation offered by Aboulfalah may be a little obscure. Since they stated, “this technique deserves to be applied in greater number,” its clarification may be valuable for those unfamiliar with the technique. We interpret their technique as follows. A needle is used to penetrate the abdominal wall just above the symphysis pubis and then transfix the uterus (anterior → posterior) in the lower uterine segment (Point A in Figures 1A,B). Then, the suture runs over the uterine fundus. This is Hayman’s simple brace suture (2). Then, importantly, the needle penetrates the abdominal wall (abdominal cavity → surface) at a site 2 cm cephalad to the initial puncture site (Point B in Figures 1A,B). The same procedure is then performed on the opposite side. The suture is tied outside the abdominal wall: lower with lower, and upper with upper sutures (Figure 1B, upper inset). By tying the sutures tightly, the uterus assumes a marked ante-flexion position and is compressed against the pubis (Figure 1B).

**Figure 1:**
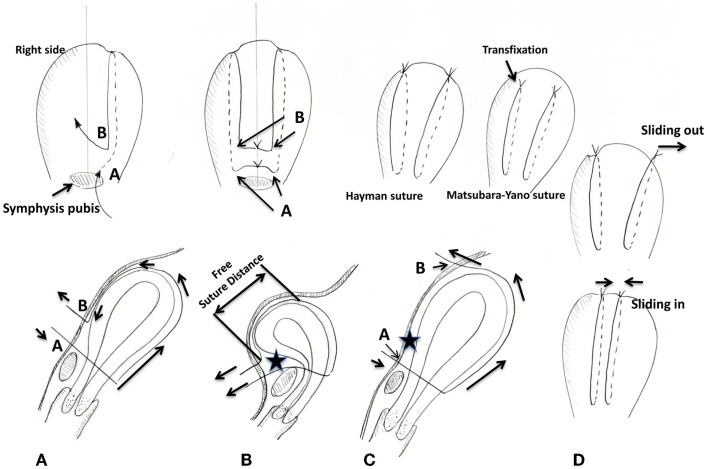
**Schematic presentation of the Aboulfalah removable uterine compression suture (A,B), our proposed concept (C), and sliding out/in of the suture (D)**. **(A)** The Aboulfalah technique. The upper inset illustrates the anterior view. **(B)** Tying the suture in the Aboulfalah technique. The suture is pulled (arrows) and tied, and, thus, the uterus assumes an anteflexed position. The suture runs freely along the anterior uterine wall. There is a space between the suture and the anterior uterine surface (star). The upper inset illustrates the anterior view. **(C)** Our proposed concept. Compared with the Aboulfalah technique [**(A,B)**, point B], point B is more cephalad. Thus, the anterior uterine wall becomes compressed against the abdominal wall. There is no space [comparing the star between this figure and **(B)**]. Upper inset shows the schema of the Hayman suture and the Matsubara–Yano (MY) suture, respectively. **(D)** The suture sliding out and sliding in. The suture tends to slide out/in, both of which result in insufficient uterine compression. Hayman referred to sliding out as “like braces off a round-shouldered man.” The MY suture prevents this sliding out/in.

We have two technical concerns: possible weaker compression and the risk of the suture sliding. Publication delay prevented Aboulfalah et al. from citing our recent article (3) in which a similar technique was proposed, although theoretical, employing the concept of a removable UCS. Figure 1C illustrates the proposed concept. The Hayman suture (upper inset left) is performed. The MY suture is also applicable, in which not only the lower uterine segment but also upper part of the uterine body is transfixed (Figure 1C upper inset right). In either technique, the suture is tied outside the abdominal wall. Regardless whether the Hayman or MY suture is employed, in a manner different from the Aboulfalah technique, the cephalad suture is placed more cephalad than in the Aboulfalah technique (comparing point B in Figure 1A vs. Figure 1C). In the Aboulfalah technique, the cephalad suture is placed more caudally on the abdominal wall. Thus, the anterior part of the uterus may not be well compressed (star in Figure 1B). This allows a space to form between the abdominal wall and the uterine anterior surface (comparing the stars in Figures 1B,C). Furthermore, compared with our technique, the suture runs freely over a longer distance (Figure 1B), and thus, the suture “sliding out” (Figure 1D upper) or “sliding in” (Figure 1D lower) may occur more frequently. As we have pointed out (2), sliding out/in sometimes occurs with the Hayman or B-Lynch sutures and preventing effective uterine compression. Thus, our technique may have (1) more compression and (2) less chance of the suture sliding out/in, but may have no hemostatic mechanism derived by “compression of the uterus against the pubis.” The inverse is true of the Aboulfalah technique. In our opinion, if the compression force is strong, uterine compression against the pubis may not necessarily be needed. This approach favors sufficient compression by the suture alone, compared with against the pubis compression.

Although theoretical, we proposed another technique for removing the suture, employing vaginal removal of the suture (3, 4). Quite recently, this concept was described by other investigators (5). Further study is needed to determine which route, abdominal or vaginal, may be better for removing the UCS. In either scenario, the procedure should be safe and easy. We believe that “removing a foreign body” is a fundamental concept in the practice of medicine (3).

The year 1997 opened a new era of PPH treatment. However, the concept of a UCS is not yet complete. The presence of various modifications of the UCS indicates that there is no “best” method for placing or removing a UCS. A removable UCS may be promising and its introduction may open a second new era of PPH treatment. Wider discussion may hasten adoption of this technique.

## Author Contributions

SM, HT, and AL meet all four criteria: (1) substantial contributions to the conception of the work, (2) drafting the work for important intellectual content, (3) final approval of the version to be published, and (4) agreement to be accountable for all aspects of the work in ensuring that questions related to the accuracy or integrity of any part of the work are appropriately investigated and resolved.

## Author’s Note

Sources of funding: none; patient anonymity: preserved; informed consent: obtained.

## Conflict of Interest Statement

The authors declare that the research was conducted in the absence of any commercial or financial relationships that could be construed as a potential conflict of interest.

## Abbreviations

MY, Matsubara–Yano; PPH, postpartum hemorrhage; UCS, uterine compression suture.
